# Case Report: The first case of primary pulmonary collision tumor comprising mixed squamous cell and glandular papilloma and glomus tumor

**DOI:** 10.3389/fonc.2022.1050220

**Published:** 2022-11-21

**Authors:** Chan Yang, Shuang Li, Zuoyu Liang, Lili Jiang

**Affiliations:** Department of Pathology, West China Hospital, Sichuan University, Chengdu, China

**Keywords:** pulmonary collision tumor, mixed squamous cell and glandular papilloma, glomus tumor, biphasic tumor, histopathology

## Abstract

A collision tumor is a rare entity, particularly if occurring in the lung. We report a case of a 57-year-old woman with a primary pulmonary collision tumor comprising mixed squamous cell and glandular papilloma (MSGP) and glomus tumor (GT). An abnormal mass was discovered in the right lung by computed tomography (CT) of the chest. A right lower lobectomy with mediastinal lymph node dissection was performed. Histological examination of the surgical specimen suggested that the lung cancer was composed of two neoplastic components. To the best of our knowledge, this is the first report of a primary pulmonary collision tumor comprising two benign tumors of different origins, which were MSGP and GT.

## Introduction

Collision tumors are defined as independent neoplasms comprising two or more distinct tumor compositions, coexisting in the same site and growing closely to one another, without any area of intermingling (1). Because of similar histology, collision tumors must be distinguished from other tumors containing two or more components. 1) A combined tumor, a neoplasm composed of two histologically and immunohistochemically distinct but intertwined cell populations coming from a common source, has a common driver mutation with environmental impact resulting in divergent morphology (2, 3). 2) Multiple primary tumors, including more than one separate neoplasm, arise in obviously different sites and/or occur at different times (4). 3) A biphenotypic tumor is defined as a tumor that consists of two cell populations, arising from a common stem cell, and that undergoes divergent differentiation with common immunohistochemical properties (5). The occurrence of collision tumors in the lung is extremely rare than in other various organ systems. Pulmonary adenosquamous carcinoma is classified as a collision tumor. By contrast, combined small-cell carcinoma, combined large-cell neuroendocrine carcinoma, and carcinosarcoma are classified as combined tumors, and pulmonary blastoma is classified as a biphasic tumor.

In this study, we reported an extremely rare primary pulmonary collision tumor comprised of two benign tumors: mixed squamous cell and glandular papilloma (MSGP) standing for epithelial element and collision tumor (GT) standing for mesenchymal element. In addition, the literature about collision tumors on PubMed was retrieved with the keywords “pulmonary/lung & collision tumor” and “bronchus/bronchioles & collision tumor.” With a literature review, we integrated and summarized the clinicopathological features, treatment, and outcome of various types of collision tumors.

## Case report

**Figure 1:**
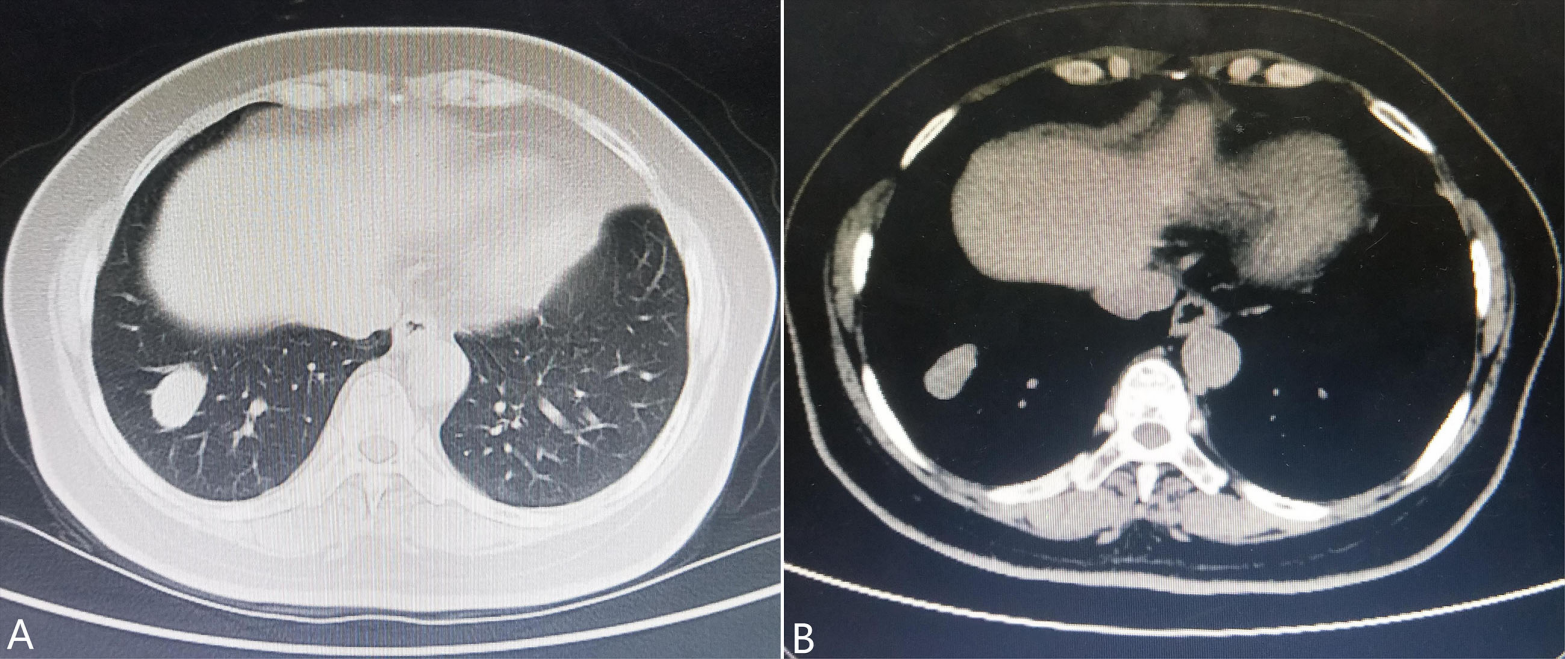
Chest computed tomography shows **(A)** a nodulous shadow located in the segmentum basale laterale of the right lower lobe measuring 2.6 cm × 1.8 cm in size with a smooth margin and **(B)** soft tissue density.

A 57-year-old woman, a non-smoker, with a medical history of type 2 diabetes mellitus was incidentally detected with a nodulous shadow on a chest radiograph obtained as part of a routine physical examination. Follow-up observation was performed and she did not receive any treatment. Three years later, computed tomography presented a 2.6-cm × 1.8-cm mass with soft tissue density and smooth margin in the lateral basal segment of the right lower lobe (**Figure 1**). The distal bronchi were slightly dilated and the wall of the bronchial lumen was thickened in the right lower lung field. Physical examination showed no evidence of lymphadenopathy or pleural effusion. No significant metabolic activity can be found in a positron emission tomography (PET)-CT scan. Except for CA242 (slightly increased), most serum tumor markers were within the normal ranges. Subsequently, a right lower lobectomy with lymph node dissection *via* video-assisted thoracoscopic surgery was performed.

**Figure 2:**
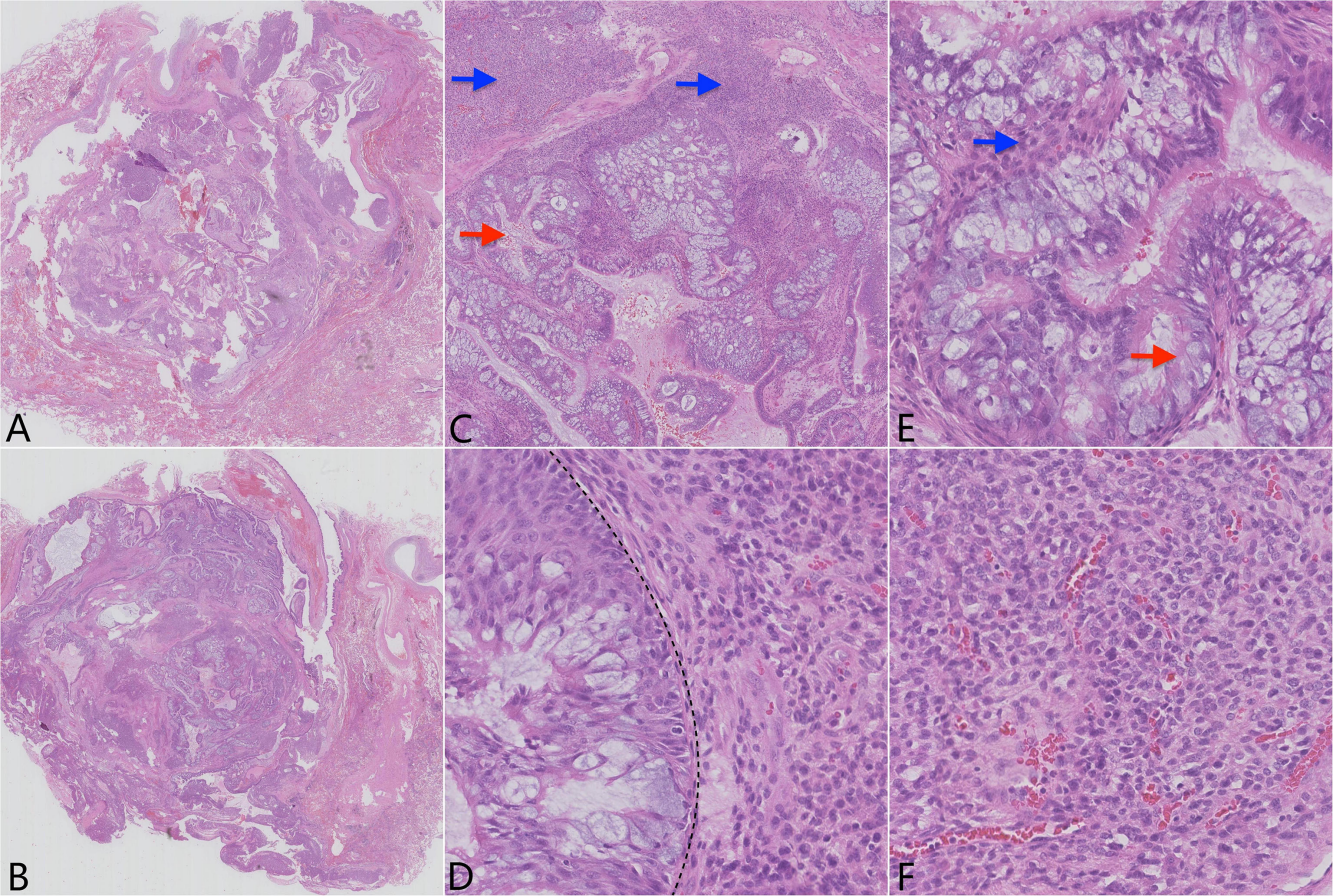
Pathological findings of the tumor show two distinct histological subtypes. **(A)** H&E of the whole slide scan showing the well-defined tumor located in the dilated bronchus or bronchiole and **(B)** focally connecting to the normal bronchial walls. **(C)** Lower power H&E (×4) reveals two distinctly demarcated tumor cell populations (red arrow: epithelial component; blue arrow: mesenchymal component). **(D)** High power H&E (×40) reveals that the two components grow closely to each other, but without a transitional zone (line). **(E)** H&E (×40) of the mixed squamous cell and glandular papilloma (red arrow: glandular component; blue arrow: squamous cell component). **(F)** H&E (×40) of the glomus tumor component.

**Figure 3:**
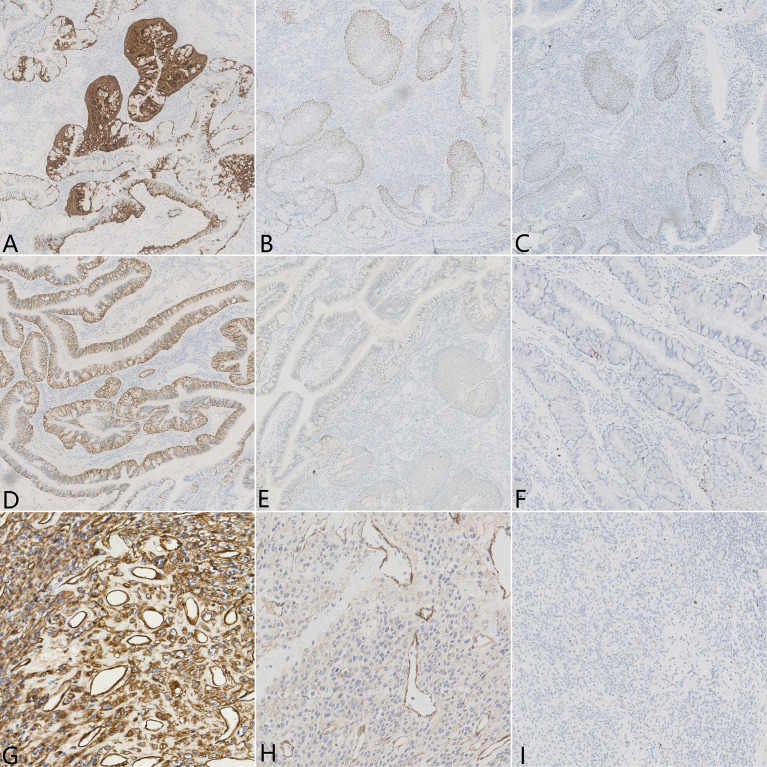
Immunohistochemical results of the pulmonary collision tumor. Epithelial element stains were positive for **(A)** CK5/6, **(B)** P63, **(C)** P40, and **(D)** CK7 but negative for **(E)** TTF-1. **(F)** Ki-67 was positive on the basal side of the areas of squamous epithelial, approximately 1%–2%. Mesenchymal element stains were diffusely positive for **(G)** collagen IV and **(H)** MSA. **(I)** The percentage of Ki-67 expression in the area of glomus tumor cells was less than 1%.

A gross examination of the resected specimen showed an ill-defined, solid gray-white nodule measuring 1.7 cm × 1.5 cm × 1.2 cm. The visceral pleura and the surgical margin were not involved. Microscopically, the tumor was a conspicuous endobronchiolar growth within the dilated bronchus or bronchiole (**Figures 2A, B**, whole slide scan). The tumor showed a well-demarcated border, which was connected to the normal bronchial walls in some areas (**Figure 2B**). At low magnification, the tumor was composed of two different histological components, including MSGP and GT (**Figure 2C**). Although the two kinds of tumor cells were close to each other, a distinct demarcation between them could still be found, there were no intertwined components, and a transitional morphology existed in the neighboring cell populations (**Figure 2D**). At high magnification, the component of MSGP showed a prominent papillary architecture with a fibrous vessel axis, lined with discrete foci of squamous and glandular epithelium. The glandular epithelium was composed of a mixture of ciliated or non-ciliated columnar cells, mucous columnar cells, and scattered goblet cells (**Figure 2E**). No significant glandular atypia, necrosis, or stromal infiltration could be found, but squamous cells showed mild atypia. The GT is composed of nests of glomus cells surrounding various vessels. The uniform tumor cells were small and round, with inconspicuous nucleoli, lightly eosinophilic cytoplasm, and sharply defined cell margins (**Figure 2F**). Mitotic figures were generally absent. All lymph nodes were negative. Immunohistochemically, the squamous component of MSGP was positive for CK5/6, P63, and P40, and the glandular component of MSGP was positive for CK7 (**Figures 3A–D**). Both components were positive for AE1/AE3 and negative for thyroid transcription factor-1 (TTF-1) (**Figure 3E**) and napsin A. Ki-67 was positive on the basal side of the areas of squamous epithelial (**Figure 3F**). GT was diffusely positive for collagen IV (**Figure 3G**) and muscle-specific actin (MSA) (**Figure 3H**), focally positive for α-smooth muscle actin (α-SMA), weakly positive for CD56, and negative for caldesmon, calponin, CD34, ERG, and STAT6. The Ki-67 index was less than 1% (**Figure 3I**). Two types of tumor cells were all focally positive for p53 and negative for S-100, SOX10, synaptophysin (Syn), and chromogranin A (CgA). Given all this, the tumor was diagnosed as a primary pulmonary collision tumor comprising MSGP and GT. There was no evidence of recurrence or metastasis after 6 months of follow-up.

## Discussion

The 57-year-old female patient, despite having a pulmonary mass, was asymptomatic during her routine hospital visits. When making a primary diagnosis, based on clinicopathological features, two types of tumors should be considered: 1) mesenchymal cystic hamartoma and 2) collision tumor.

Mesenchymal cystic hamartoma (MCH) is a rare lung disease with an indolent clinical course. It is composed of primitive mesenchymal cells that gradually promote the formation of nodules and cysts (6). Histologically, these cystic spaces are lined with normal respiratory epithelium and surrounded by a proliferation of immature mesenchymal cells (6). On immunohistochemistry, the epithelial cells are diffusely positive for AE1/AE3 and TTF-1; the mesenchymal cells are diffusely positive for vimentin, with a low Ki-67 index of about 1%. It is noteworthy that the mesenchymal cells are negative for most specifically differentiated markers such as CD31, CD34, desmin, SMA, myoglobin, S-100, D2-40, etc. (7).

Immunohistochemistry of the tumor in our case revealed the following results: mesenchymal cells were diffusely positive for collagen IV, MSA, and CD56 and focally positive for SMA, which confirmed that the mesenchymal neoplasm is similar to GT. Meanwhile, the results excluded the possibility of MCH. On the other hand, epithelial cells were positive for AE1/AE3, CK7, CK5/6, P63, and P40, indicating MSGP, which is one of the solitary endobronchial papillomas. Negative staining of TTF-1 in epithelial cells also excluded the diagnosis of MCH.

Except for the combined tumors, multiple primary tumors, and biphenotypic tumors mentioned in the *Introduction*, only seven primary pulmonary collision tumor cases have been reported in the literature. We can further group these collision tumors according to their composition: four collision tumors, namely, malignant melanoma colliding with adenocarcinoma (8), large-cell carcinoma colliding with adenocarcinoma (9), adenocarcinoma colliding with typical carcinoid (10), and lymphoepithelioma-like carcinoma colliding with adenocarcinoma (11), are comprised of two different epithelial elements (**Table 1**); and three collision tumors, namely, squamous cell carcinoma colliding with T-cell lymphoma (12), epithelioid hemangioendothelioma colliding with bronchioloalveolar carcinoma (13), and carcinoid colliding with high-grade spindle-cell sarcoma (14), are comprised of epithelial and mesenchymal elements (**Table 2**). As we all know, a collision tumor composed of two benign entities has not been reported in the literature.

**Table 1:** Summary of published pulmonary collision tumors comprising two different epithelial elements found in the literature.

Case no.	Year	Author	Age/sex	Location	Maximum diameter (cm)	Components of pulmonary collision tumor		Imaging findings	Treatment	Outcome (follow-up time)
						Epithelial element	Epithelial element			
1	1993	T. Ueyama (8)	61/F	Left lower	4	Malignant melanoma	Adenocarcinoma	Irregular opaque shadow	Chemotherapy was performed with DTIC (dimethyl triazeno imidazole carboxamide) and interferon	Death (12 months)
2	2003	Shoji Nakata (9)	53/M	Right upper	4.2	Large cell carcinoma	Adenocarcinoma	Dumb bell-shaped mass	Right upper lobectomy with a mediastinal lymph node dissection	No evidence of recurrence (16 months)
3	2014	Kamal K. S. Abb (10)	70/F	Left upper	1.3	Typical carcinoid	Adenocarcinoma	Hazy density	Right upper and middle lobectomies with a mediastinal lymph node dissection	Not available
4	2015	Jammy Kin Iong Chan (11)	59/F	Left upper	4	Lymphoepithelioma-like carcinoma	Adenocarcinoma	Dumbbell-like mass	Left upper lung and associated hilar lymph node dissection and an EP regimen for four cycles, and then targeted therapy with an EGFR-tyrosine kinase inhibitor	Survival (12 months)

**Table 2:** Summary of published pulmonary collision tumors comprising epithelial and mesenchymal elements found in the literature.

Case no.	Year	Author	Age/sex	Location	Maximum diameter (cm)	Components of pulmonary collision tumor		Imaging findings	Treatment	Outcome (follow-up time)
						Epithelial element	Mesenchymal element			
1	1999	Osamu Kawashima (12)	71/F	Right lower	4.5	Squamous cell carcinoma	T-cell lymphoma	Mass	Right lower lobectomy with lymph node dissection and systemic chemotherapy for malignant lymphoma	Death (7 months)
2	2003	Takahashi K (13)	59/F	Left upper	1.4	Bronchioloalveolar carcinoma	Epithelioid hemangioendothelioma	Ill-defined shadow	Complete lingulectomy	No evidence of recurrence (13 years)
3	2011	Corinne Liu (14)	54/F	Right upper	4.5	Carcinoid	High-grade spindle-cell sarcoma	Well-defined mass	Right upper lobectomy with lymph node dissection	Bone metastasis (18 months)

GTs are defined as benign tumors comprising neuromyoarterial glomera or glomus bodies originating from modified smooth muscle cells and surrounding arteriovenous anastomosis (15), which account for less than 2% of soft tissue tumors (16). These tumors commonly occur in subungual areas and deep dermis of the extremities where glomus bodies abound and, occasionally, in areas where glomus bodies are spare or absent, such as the gastrointestinal tract (17), bladder (18), bone (19), eyelid (20), cervix (21), ovary (22), thyroid (23), and thorax (24, 25). GTs rarely originated from the respiratory system, and the trachea is the more common site among them, with 77 cases reported to date (26). Originating from the lung is extremely uncommon, with less than 50 cases (27). Clinically, the symptoms of bronchial or tracheal GTs were commonly associated with airway irritation, but GTs occurring in the peripheral lung were generally asymptomatic (28). GTs of the respiratory tract generally occurred in middle-aged individuals (29) with no obvious sex predilection, and the maximum diameter ranged from 0.7 to 9.5 cm (15, 30). Morphological similarity may misdiagnose primary bronchopulmonary GT as carcinoid, hemangiopericytoma, paraganglioma, sugar tumor, or sclerosing pneumocytoma. Immunohistochemically, typical GTs are positive for SMA, MSA, h-caldesmon, calponin, and collagen IV. CD34 and desmin are also expressed focally in some cases, while cytokeratin, EMA, bcl−2, TTF-1, HMB45, CD117, LCA, CgA, CD31, and S-100 are invariably negative (31). Among primary lung tumors, GTs are often misdiagnosed as carcinoid for their cytomorphological features. On immunohistochemical analysis, a carcinoid shows positivity for neuroendocrine markers, such as CD56, Syn, and CgA, whereas a GT shows the opposite.

Sclerosing hemangioma is composed of cubic epithelial cells and circular stromal cells, and all these cells express TTF-1 and EMA. Paraganglioma is positive for the S-100 protein and neuroendocrine markers. A sugar tumor is diffusely positive for HMB45, which is conducive to differentiating it from GT. Occasionally, epithelioid leiomyoma can show similar histologic morphology with GT and can also express SMA. However, epithelioid leiomyoma expresses other smooth muscle markers including desmin and caldesmon.

Based on the World Health Organization’s (2013) classification of soft tissue tumors, GTs are divided into benign GTs, GTs with uncertain malignant potential, and malignant GTs (32). Previous retrospective studies on primary bronchopulmonary GTs found that about 74% of cases are benign, followed by malignant cases, accounting for approximately 21%, and cases with uncertain malignant potential account for approximately 5% (15, 30). Given that malignant GTs have aggressive behavior, with up to 38% of patients developing metastasis (31) and the possibility of local recurrence for incomplete extirpation, radical surgical resection is still essential for long-term prognosis.

Solitary endobronchial papillomas are rare benign pulmonary tumors occurring in the bronchus, and they were identified in 1998, including squamous cell papilloma, glandular papilloma, and mixed squamous cell and glandular papilloma (33). The mixed entity is the rarest, showing a mixture of glandular and squamous epithelium. Each epithelial type should constitute more than one-third of the tumor (34). Occasionally, glandular epithelium can also grow with a micropapillary pattern and drop into alveolar lumens, which is easy to be misdiagnosed as micropapillary adenocarcinoma. Misdiagnosis of mucinous adenocarcinoma usually happens when mucus cell components are rich. Adenocarcinoma cells have significant atypia, without basal cells, leading to negative staining for p40 and CK5/6.

The case we presented here where GT collided with MSGP in the lung has never been reported in the literature. As with other pulmonary collision tumors that had been described, with no characteristic clinical features and imaging findings, the primary diagnosis is difficult (35), which mostly depends on the different neoplasms of the collision tumor, and the definitive diagnosis mainly relies on the pathological diagnosis.

The exact pathogenesis behind pulmonary collision tumor remains unknown. A most widely accepted theory is that each component of the collision tumor has different behavioral, histological, and genetic features, revealing their intratumoral heterogenicity and their different tumorigenesis (5, 36). Four main hypotheses have been proposed in the literature: 1) the two different types of tumors are accidentally colliding in one location (37); 2) the carcinogenic stimulus causes an increased chance of arising the simultaneous separate neoplasms at the same site (8); 3) one neoplasm induces the development of another different neoplasm through paracrine effects changing the microenvironment (38); and 4) single-cell gene mutation leads genetically homogeneous clonal cells to differentiate into histologically separate tumor cells due to the heterogeneity of the gene phenotypes (39). In our case, GT and MSGP abutted each other, but two different origins can still be recognized, and there were no mutual migration phenomena or transitional pattern histomorphologically. Furthermore, the two kinds of tumor cells of pulmonary collision tumor had different immunophenotypes demonstrating that each of them possessed different molecular properties and cytogenetics, suggesting their different tumorigenesis. Considering the clinicopathological results of our case, the case was finally diagnosed as pulmonary collision tumor consisting of MSGP and GT. With the review of CT, the density of the lesion was heterogeneous, suggesting its different cell components. Treatment and prognosis largely depend on the independent biological behavior of different histological subtypes of pulmonary collision tumor.

As the first case of primary pulmonary collision tumor comprising two benign tumors, the prognosis of this subtype has not been clearly clarified. To date, no evidence of recurrence or metastasis has been shown by the patient. We will continue to focus on the prognosis of this patient in the future. Given the rare incidence of collision tumor, the inadequate attention given to it, and the insufficient research conducted about it, further studies and in-depth research are needed to elucidate its mechanism of tumorigenesis and biological behavior.

## Data availability statement

The original contributions presented in the study are included in the article/Supplementary Material. Further inquiries can be directed to the corresponding author.

## Ethics statement

The studies involving human participants were reviewed and approved by Biomedical Research Ethics Committee of West China Hospital. The patient provided her written informed consent to participate in this study. Written informed consent was obtained from the individual(s) for the publication of any potentially identifiable images or data included in this article.

## Author contributions

LJ contributed to the conception and design of the work. CY collected the data and wrote the original draft. SL contributed to the interpretation of data. ZL revised the manuscript. All authors approved the final version of the manuscript.

## Funding

This study was funded by the Project for Disciplines of Excellence-Clinical Research Incubation Project, West China Hospital, Sichuan University (No. 2019HXFH002).

## Conflict of interest

The authors declare that the research was conducted in the absence of any commercial or financial relationships that could be construed as a potential conflict of interest.

## Publisher’s note

All claims expressed in this article are solely those of the authors and do not necessarily represent those of their affiliated organizations, or those of the publisher, the editors and the reviewers. Any product that may be evaluated in this article, or claim that may be made by its manufacturer, is not guaranteed or endorsed by the publisher.
